# Clinical Mastitis in Small Ruminants Referred to a Veterinary Teaching Hospital: 23 Cases

**DOI:** 10.3390/microorganisms13071512

**Published:** 2025-06-28

**Authors:** Gabriel Inácio Brito, Liz de Albuquerque Cerqueira, Simone Perecmanis, José Renato Junqueira Borges, Márcio Botelho de Castro, Antonio Carlos Lopes Câmara

**Affiliations:** 1 Large Animal Veterinary Teaching Hospital, College of Agronomy and Veterinary Medicine, Universidade de Brasília, Brasília 70636-200, DF, Brazil; gabritoinacio29@gmail.com (G.I.B.); jrborges@unb.br (J.R.J.B.); 2 Veterinary Pathology and Forensic Laboratory, College of Agronomy and Veterinary Medicine, Universidade de Brasília, Brasília 70910-900, DF, Brazil; lizcerqueira@hotmail.com (L.d.A.C.); mbcastro@unb.br (M.B.d.C.); 3 Veterinary Microbiology Laboratory, College of Agronomy and Veterinary Medicine, Universidade de Brasília, Brasília 70910-900, DF, Brazil; perecmaniss@unb.br

**Keywords:** intramammary infection, goat, mastectomy, sheep, systemic disturbances

## Abstract

Clinical mastitis in small ruminants is usually seen with an incidence of less than 5% and most cases, especially with hyperacute evolution, are not referred for hospital care. During the 5-year survey, 16 goats and 7 sheep, totaling 23 small ruminants, met the inclusion criteria with a definitive diagnosis of clinical mastitis. Clinical signs ranged greatly among cases, varying from septic state in hyperacute cases, and enlarged, pendulous udder associated with chronic pain and abnormal gait in chronic cases. Microbiological culture revealed a wide array of bacterial pathogens, including *Staphylococcus aureus*, *Escherichia coli*, *Streptococcus* spp., and *Pasteurella* spp. In vitro antimicrobial susceptibility profiles varied greatly among bacteria isolates, ranging from sensitive to all tested antimicrobials to a multi-resistant profile. Pathological features included hyperemia and dark red areas of necrosis in the skin, marked hyperemia of the affected gland at the cut surface, lactiferous ducts and gland cisterns filled by cloudy or suppurative fluid, abscesses, and hardness of the mammary gland parenchyma. This retrospective study highlights the multifactorial nature and clinical variability of mastitis in small ruminants, demonstrating its significant impact on animal health, welfare, and production.

## 1. Introduction

Mastitis is considered one of the most important diseases affecting domestic animals and is caused by several etiologic agents. The disease is economically relevant for sheep and goat dairy farmers due to the drop in milk production, premature culling of the dams, treatment costs, reduced offspring performance, and adverse influences on the welfare of affected animals [1,2,3].

The most common form of mastitis in sheep and goats is subclinical mastitis, with a prevalence ranging from 5 to 30% and occasionally reaching 50% in some flocks [4]. Clinical mastitis in small ruminants is usually seen with an incidence of less than 5% and most often occurs during the first third of lactation [5]. Nevertheless, mastitis outbreaks of bacterial etiology may be a prominent health problem affecting dairy sheep farms in some countries [1,2]. Early detection of mastitis during the subclinical phase is crucial for preventing progression to acute or chronic stages, reducing animal losses and minimizing economic impact [6]. Therefore, preventive measures such as the California Mastitis Test, monitoring somatic cell counts in milk, and maintaining udder and milker hygiene to enable early detection is essential [7].

The term mastitis refers to the presence of an inflammatory process in the mammary gland; typically, a bacterial infection triggers an inflammatory reaction, resulting in the development of variable clinical signs. The clinical syndrome can follow a variable course, ranging from hyperacute to chronic. Depending on the clinical course, mastitis can be conventionally differentiated into hyperacute, presenting a serious udder inflammation accompanied by an evident systemic response; acute, when severe udder inflammation is present but there is no systemic involvement; and chronic, when there is no systemic involvement and only fibrous lesions, sometimes poorly detectable, are present [2,8]. However, classification can be challenging, as some cases of chronic mastitis may also present with systemic signs, such as chronic pain, progressive weight loss, and anemia [8].

Additionally, as the goat and sheep dairy industry continues to grow substantially each year, it is essential to investigate whether antimicrobial-resistant bacteria are present in the milk of clinically affected animals, which is essential for both human and animal medicine, as part of the One Health perspective [9]. Considering this scenario, the present study aimed to present epidemiological, clinical, laboratory, microbiological, and pathological features of clinical mastitis in 23 small ruminants referred to a Veterinary Teaching Hospital.

## 2. Materials and Methods

A 5-years retrospective survey (from January 2019 to December 2024) was conducted in the records of small ruminants referred to the Large Animal Veterinary Teaching Hospital, Universidade de Brasília, located in Brasília, Distrito Federal, Midwestern Brazil. The criteria for inclusion were a conclusive diagnosis based on a combination of epidemiological, clinical, laboratory, and/or pathological evaluations.

Epidemiological aspects encompassed species (goats or sheep), breed, and age. Clinical aspects (clinical evolution, results of physical examination, affected mammary gland, treatment, outcome, and sequelae) were extracted from veterinary reports documented in the medical records. Clinical mastitis cases were classified as hyperacute (1–48 h), acute (48 to 96 h), and chronic (>7 days) according to the duration of the disease.

After the initial clinical examination, blood samples were collected by jugular vein puncture for hematological and biochemical analysis. Pathological evaluation of tissue samples obtained by surgical excision (radical or unilateral mastectomy) was performed, and dead animals (spontaneous death or humanely euthanized) were submitted for necropsy. Tissues were fixed in 10% buffered formalin, routinely processed, paraffin-embedded, sectioned at 5 μm thick, and stained with hematoxylin and eosin (H&E) for evaluation under an optical microscope.

Milk samples were collected aseptically from the affected mammary gland during the initial physical examination and transported to the laboratory. Immediately after, the fresh samples were seeded on 8% ovine blood agar medium (Sigma-Aldrich, Darmstadt, Germany) and MacConkey agar (Neogen Corporation, São Paulo, Brazil) medium at 37 °C in aerobiosis for 24–72 h. Simultaneously, the same samples were subjected to microaerophiles (5% CO_2_) and anaerobic cultures on sheep blood agar and incubated at 37 °C for 120 h. Isolates were identified based on the standard bacteriological approaches, such as colony morphology, pyocyanin pigment production, Gram staining, growth at 44 °C, and biochemical tests: oxidase, catalase, nitrate reduction, indole, methyl red, Voges–Proskauer, citrate utilization, and glucose fermentation according to Quinn et al. [10].

Isolates were subjected to in vitro susceptibility testing (disk diffusion method), according to the Clinical Laboratory Standards Institute (CLSI guidelines [11]) using 12 antimicrobials belonging to eight groups, as follows: (1) aminoglycosides (gentamicin 10 μg), (2) beta-lactams (amoxicillin 10 μg; ampicillin 10 μg; penicillin 10 μg), (3) cephalosporins (ceftiofur 30 μg); (4) fluoroquinolones (enrofloxacin 5 μg, ciprofloxacin 5 μg, norfloxacin 5 μg), (5) macrolides (azithromycin 15 μg); (6) florfenicol 10 μg, (7) sulfonamides (sulfamethoxazole 25 μg), and (8) tetracyclines (tetracycline, 30 μg).

## 3. Results

**Figure 1:**
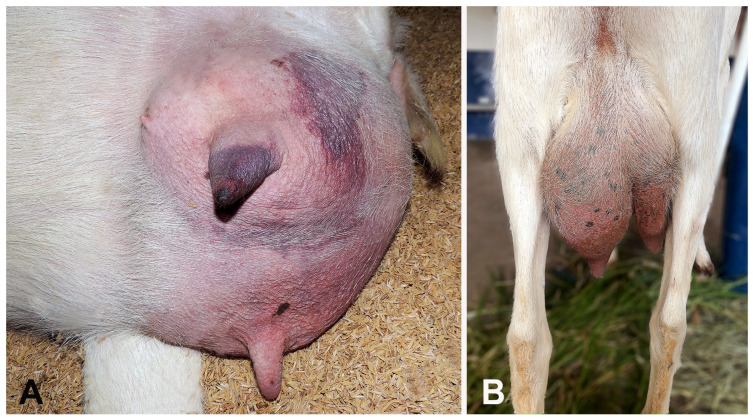
Clinical aspects of the affected mammary glands of small ruminants with mastitis. (**A**) Goat. Redness and red-black areas in the skin of the teat and udder. (**B**) Goat. Asymmetry of the udder due to unilateral enlargement of the left gland.

During the 5-years survey, 16 goats and seven sheep, totaling 23 small ruminants, met the inclusion criteria with a definitive diagnosis of clinical mastitis. Epidemiological data and clinical aspects are summarized in Table 1. The age of the affected females ranged from 12 to 72 months (mean: 43 months). The duration of the disease on the farms until clinical evaluation ranged from 12 h to 5 months. According to this data, clinical mastitis was classified as hyperacute (*n* = 11), acute (*n* = 3), or chronic (*n* = 9) cases. In the former group, the most important clinical signs included depression, anorexia, tachycardia, ruminal and intestinal hypomotility, and mammary glands with dark-blueish discoloration. Manipulating the affected mammary gland during milking elicited pain and yielded pinkish to dark-reddish milk coloration with a fetid odor. Physical examination in acute cases revealed mild clinical signs, such as hyporexia and hyperthermia, and most mammary glands presented reddish skin and red-black discoloration areas (Figure 1A), asymmetry of the udder (Figure 1B), and were hot at the touch. The most important clinical signs in chronic cases included loss of body condition score, pale ocular mucous membranes, and alterations on the mammary glands consisting of hardened consistency (fibrosis), lumpy areas (abscesses), and milking with varying degrees of clots to purulent discharge. In some cases, the enlarged udder caused abnormal gait. Hospitalization days ranged from 1 to 87 days (mean: 22.3 days).

Laboratorial data (blood tests and biochemical analysis) are presented in Table 2. One doe (Case 5) died shortly after initial clinical examination, and blood samples were not collected. In most cases, does with hyperacute mastitis presented leucopenia (*n* = 5), leukocytosis by neutrophilia (*n* = 2), or just an inversion in the neutrophils–lymphocytes ratio (*n* = 2). Serum biochemistry revealed hypoproteinemia by hypoalbuminemia in most cases, whilst increased creatinine levels was present in two animals (Case 3 and 4). Leucocytosis by neutrophilia was also present in acute mastitis cases associated to hyperfibrinogenemia. The most relevant alterations in chronic cases consisted of anemia, hypoalbuminemia, and hyperglobulinemia. Only two animals presented hematological and biochemical values within the reference range [12,13].

Microbiological assays and in vitro antimicrobial susceptibility profiles of milk samples were performed in 13 small ruminants (10 goats and 3 sheep) with clinical mastitis (Table 3). In hyperacute cases (*n* = 8) presenting the most severe clinical disturbances, the isolated microorganisms included *Escherichia coli* (*n* = 2), *Staphylococcus aureus* (*n* = 2), *Streptococcus* sp. (*n* = 1), and *Pasteurella* sp. (*n* = 1), whilst two cases presented no bacterial growth. One case with acute evolution (Case 18) yielded *S. aureus* on both mammary glands, but the bacteria presented different susceptibility profiles. Chronic cases included Case 6, which offered a mixed bacterial growth (*E. coli* and *S. aureus*); Case 14, with one bacteria species affecting each mammary gland (*E. coli* on right and *S. aureus* on left mammary gland); and Cases 7 and 19 with no bacterial growth. In vitro antimicrobial susceptibility profiles varied greatly among bacteria isolates, ranging from sensitive to all tested antimicrobials (Case 4) to a multi-resistant profile (Case 21).

Of the 23 small ruminants with clinical mastitis, 17 (73.9%) received hospital discharge, and 6 (26.4%) died. Euthanasia was performed due to welfare issues and poor prognosis in two animals (Case 6 and 7), and spontaneous death occurred in four small ruminants (Case 3, 4, 5, and 11) despite intensive care. Therefore, pathological evaluation was performed on six dead small ruminants (four goats and two sheep) and also on seven incisional biopsy samples from radical (*n* = 6) and unilateral (*n* = 1) mastectomy cases.

**Figure 2:**
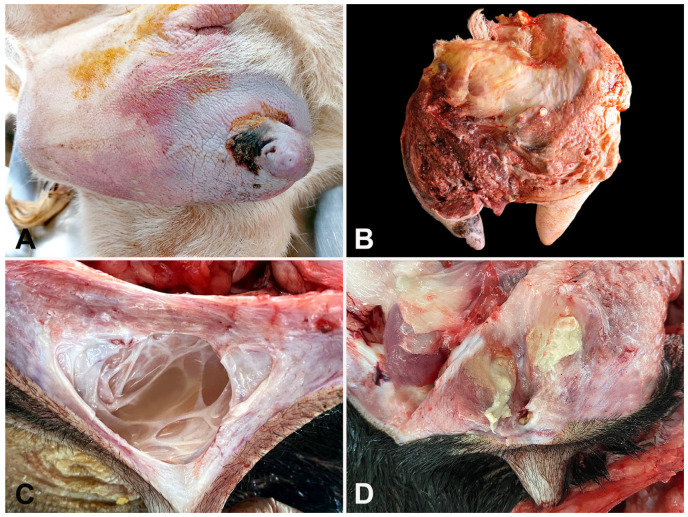
Gross pathological alterations in the mammary tissues of small ruminants with mastitis. (**A**) Goat. Hyperemia in the skin of the udder and a dark area of necrosis affecting the teat. (**B**) Goat. Marked hyperemia of the affected parenchyma. (**C**) Sheep. Lactiferous sinus filled with a white-cloudy inflammatory fluid. (**D**) Sheep. An abscess within the parenchyma close to the teat cistern.

**Figure 3:**
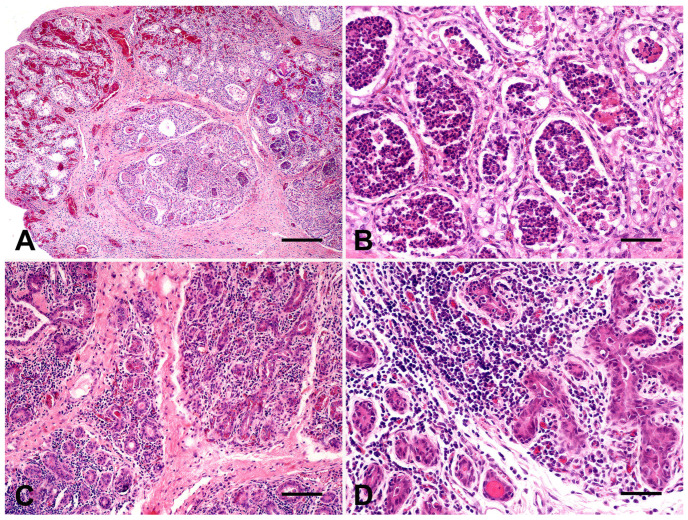
Histological findings in the mammary tissues of small ruminants with mastitis. (**A**) Goat. Marked hyperemia of interstitial vessels in the mammary lobes and inflammatory infiltrate filling alveoli (H&E, bar = 250 μm). (**B**) Goat. Alveolar epithelial degeneration, necrosis, and numerous neutrophils fill alveoli (H&E, bar = 50 μm). (**C**) Sheep. Interstitial mononuclear inflammatory infiltrate within mammary lobes (H&E, bar = 100 μm). (**D**) Severe lymphocytic inflammatory infiltration surrounding mammary alveoli (H&E, bar = 25 μm).

Gross and pathological findings observed in affected sheep and goats were hallmarks for diagnosing mastitis in this study. Affected animals frequently showed hyperemia and dark red areas of necrosis in the skin (Figure 2A), marked hyperemia of the affected gland at the cut surface (Figure 2A), lactiferous ducts and gland cisterns filled by cloudy (Figure 2C) or suppurative fluid, abscesses (Figure 2D), and hardness of the mammary gland parenchyma. Microscopically, acute mastitis was characterized by remarkable hyperemia, hemorrhagic foci, alveolar epithelial degeneration, and inflammatory neutrophilic infiltration (Figure 3A), mainly within glandular alveoli (Figure 3B). Chronic cases showed abscesses in the mammary parenchyma, numerous foci of lymphocytic or lymphoplasmacytic inflammatory infiltrate within lobes (Figure 3C) and surrounding alveoli (Figure 3D), and scattered foci of fibrosis.

## 4. Discussion

Mastitis is considered a multifactorial disease, implying the possible involvement of multiple risk factors, which include environmental factors, nutrition, viral infections, stage of lactation, lactation number, udder shape, teat conformation, milking technique, and equipment hygiene [14,15]. Herein, all cases in goats (*n* = 16) occurred in Saanen or crossbred does from milk flocks under intensive management submitted to manual or mechanical milking methods. In these cases, it is possible to hypothesize that poor management practices, such as the use of unsuitable milking machines and/or inadequate training of milkers in correct milking techniques, may lead to incomplete milk-out or overmilking [16]. These, along with poor udder conformation, may lead to excessive machine stripping, all of which are risk factors for teat damage and mastitis [15]. Regarding sheep, all ewes were crossbred hair sheep from semi-extensive meat production flocks with suckling lambs. Therefore, in sheep and goats, risk factors such as crowded conditions and decubitus during rumination and sleep increase the udder’s exposure to environmental microbial flora, which, in poor hygienic conditions, may increase the risk of exposure to environmental pathogens [2].

This study provides insight into clinical mastitis in small ruminants referred to a Veterinary Hospital, reiterating that the disease is life-threatening. The severe systemic disturbances observed, especially in hyperacute cases, result from the liberation of bacteria, pathogen-associated molecular patterns (PAMPs), and damage-associated molecular patterns (DAMPs) into the circulation during the septic state. The profound systemic inflammatory effects can lead to remote organ dysfunction and the development of multi-organ dysfunction syndrome (MODS). Typical organs involved in MODS include, but are not limited to, the heart, lungs, kidneys, and liver [17]. Additionally, disrupting the blood–milk barrier in cases of severe mastitis facilitates the passage of bacteria from milk into the bloodstream, thereby enhancing bacteremia [18]. Therefore, implementing protocols to decrease mastitis incidence may also be considered a welfare issue [14].

Clinical mastitis treatment includes broad-spectrum antibiotics, non-steroidal anti-inflammatory agents, palliative treatments (intravenous fluid therapy or oral hydration), and sometimes surgery (teat amputation, pudendal artery ligation, or mastectomy) [1,14,15,19]. Ideally, cultures should be obtained before the initiation of antimicrobial therapy [19], but in most instances, especially when dealing with hyperacute cases, treatment decisions have already been made before the results of antibiotic sensitivity testing become available [14,19]. In our study, four hyperacute cases that evolved to death presented antimicrobial resistance to the chosen antimicrobial when antibiotic sensitivity tests were available. Furthermore, a Saanen doe that presented a multidrug-resistant bacterial strain (Case 21) only improved with the association of an intermediate antibiotic in the sensitivity test (the only available for animal use) associated with unilateral mastectomy. These features underscore the need for aggressive treatment of hyperacute cases due to the risk of rapid deterioration, and that determining sensitivity patterns may help inform treatment decisions for future cases [13,18]. In two cases, severe damage to the mammary parenchyma caused by bacterial infection resulted in hardened mammary glands as a sequelae of clinical treatment, leading to decreased milk production and an increased future culling rate for the does and ewes [2,14,15,18].

Mastectomies were most commonly performed to resolve clinical signs associated with chronic mastitis and an enlarged, pendulous udder in the small ruminants in this study. Both conditions resulted in chronic pain and affected the quality of life, manifesting as abnormal gait, low feed intake, anemia, and weight loss, as previously reported [14,15,19,20]. After mastectomy, appetite, gait, and weight improved in all treated animals, with hospital discharges resulting in no sequelae. Despite this, the hospitalization period and surgery costs may limit mastectomies to high-value genetic animals or pet ruminants.

In this study, gross and microscopic findings were crucial in highlighting the severe suppurative inflammation, hyperemia, and hemorrhages in the mammary gland parenchyma of goats and sheep with acute mastitis, as previously described [8,20]. The majority of bacteria identified in the udders of affected animals are commonly associated with marked neutrophilic inflammation and tissue necrosis in small ruminants with mastitis [8]. As observed here, chronic mastitis is typically characterized by abscess formation, mononuclear interstitial inflammation, and fibrosis, often caused by the same pathogens in sheep and goats worldwide [8,19]. However, pathological investigations of mastitis in small ruminants remain limited, and further morphological characterization in relation to specific pathogens is still needed.

Microbiological assays in the present study revealed *S. aureus* affecting five goats and one ewe, resulting in 50% (6/12) of cultured milk samples testing positive. *S. aureus* is considered the most commonly identified cause of clinical mastitis in dairy does, and it is estimated that close to 50% of goat herds are infected with this organism [1,5,14,15], as observed herein. Additionally, a recent study in ewes highlights that, despite sampling after the regular post-milking cleaning of parlors and teatcups, staphylococci colonization still occurred [21]. The environmental group of pathogens was isolated in five cases, including pure cultures of *E. coli* obtained in two does with hyperacute mastitis. Although coliform mastitis appears to be much less common than other mastitis pathogens [15], accounting for between 1.4% and 14.2% of cases [4,7,19], it most often causes severe clinical disturbances. Other organisms that have been implicated include coagulase-negative staphylococci, *Enterobacter* spp., *Pseudomonas* spp., *Trueperella pyogenes*, *Corynebacterium pseudotuberculosis*, *Listeria monocytogenes*, *Bacillus* spp., mycoplasmas, and fungal organisms [2,3,4,5,14,15,19]. Over the last few decades, the year-by-year increase in antimicrobial resistance has become one of the most pressing global threats in both the animal and human health sectors [9,22,23]. Therefore, the microbiological isolation of multi-resistant bacterial strain raises concerns about the indiscriminate use of antibiotics in small ruminant practice, especially on milk-producing farms.

In four mastitis cases, microbiological culture resulted in no growth (Case 1, 7, 12, and 19). Since these were isolated mastitis cases in each flock, and three animals responded well to antimicrobial treatment, no further investigation was conducted to determine the etiological agent. Fortunately, *Mycoplasma* mastitis cases are uncommon in Brazil [24], and seems to be rare in our hospital routine, but since no molecular testing was performed herein, this possibility could not be dismissed. In contrast, a recent study showed that incurable mastitis in goats was associated to *M. agalactiae* infection in 59% of cases [23]. Thereby, the use of ancillary diagnostics tests, such as molecular techniques, are important to enhance diagnostic accuracy, characterize antimicrobial resistance and virulence genes of bacterial pathogens of clinical mastitis in livestock [25].

The main limitations of this study were the small sample size, lack of standardized microbiological protocols, the absence of statistical analysis, and limited examination of risk factors. Further studies are needed to address these shortcomings and produce more comprehensive and broadly applicable findings. Nevertheless, all findings reported herein are important data on clinical mastitis in small ruminants from real field conditions, since most cases, especially the hyperacute cases, are not referred for hospital care. Therefore, this retrospective study provides essential knowledge on clinical signs, laboratory and pathological features of this life-threatening condition.

## 5. Conclusions

This retrospective study highlights the multifactorial nature and clinical variability of mastitis in small ruminants, demonstrating its significant impact on animal health, welfare, and production. Clinical mastitis was classified as hyperacute, acute, or chronic, with substantial variation in disease progression and clinical presentation. Microbiological culture revealed a wide array of bacterial pathogens, including *Staphylococcus aureus*, *Escherichia coli*, *Streptococcus* spp., and *Pasteurella* spp. Several isolates showed resistance to multiple antimicrobials, reinforcing the importance of judicious antimicrobial use and susceptibility testing in treatment planning. Despite intensive care and surgical interventions, the mortality rate remained considerable, underscoring the disease’s severity and the challenges in managing advanced cases. These findings emphasize the need for early diagnosis, appropriate therapeutic strategies, and preventive measures to minimize the incidence and severity of clinical mastitis in small ruminants, especially within the growing dairy goat and sheep industries. Given the serious consequences of clinical mastitis in small ruminants, it is essential to regularly implement preventive measures on farms, such as the California Mastitis Test, monitoring somatic cell counts in milk, and maintaining udder and milker hygiene, to enable early detection during the subclinical phase.

## Figures and Tables

**Table 1:** Epidemiological data and clinical aspects from 23 small ruminants (16 goats and 7 sheep) with clinical mastitis.

Case	Specie	Breed	Age	Rearing System	Milking Method	Clinical Evolution	Affected Mammary Gland	Type of Mastitis	Treatment	Hospitalization Days	Outcome	Sequelae
1	Goat	Crossbred	4 y	Intensive	Manual	48 h	Right	Hyperacute	Clinical	9	Discharged	Rigid MG
2	Sheep	Crossbred	4 y	Extensive	None	45 days	Both	Chronic	Surgical	15	Discharged	None
3	Goat	Saanen	2 y	Intensive	Mechanical	24 h	Both	Hyperacute	Clinical	2	Death	-
4	Goat	Saanen	1 y	Intensive	Mechanical	24 h	Left	Hyperacute	Clinical	1	Death	-
5	Goat	Saanen	NAD	Intensive	Mechanical	24 h	Right	Hyperacute	Clinical	1	Death	-
6	Sheep	Crossbred	2 y	Extensive	None	37 days	Both	Chronic	Clinical	4	Euthanasia	-
7	Sheep	Crossbred	NAD	Extensive	None	8 days	Both	Chronic	Clinical	4	Euthanasia	-
8	Sheep	Crossbred	3 y	Extensive	None	96 h	Both	Acute	Surgical	60	Discharged	None
9	Sheep	Crossbred	2 y	Extensive	None	96 h	Both	Acute	Clinical	38	Discharged	Rigid MG
10	Goat	Saanen	5 y	Intensive	Manual	153 days	Both	Chronic	Surgical	58	Discharged	None
11	Goat	Saanen	4 y	Intensive	Mechanical	24 h	Both	Hyperacute	Clinical	1	Death	-
12	Goat	Saanen	6 y	Intensive	Mechanical	12 h	Right	Hyperacute	Clinical	8	Discharged	None
13	Goat	Saanen	3 y	Intensive	Mechanical	12 h	Both	Hyperacute	Clinical	10	Discharged	NAD
14	Sheep	Crossbred	5 y	Extensive	None	61 days	Both	Chronic	Surgical	22	Discharged	None
15	Sheep	Crossbred	NAD	Extensive	None	158 days	Left	Chronic	Clinical	69	Discharged	Rigid MG
16	Goat	Crossbred	5 y	Intensive	Mechanical	12 h	Both	Hyperacute	Clinical	5	Discharged	None
17	Goat	Saanen	3 y	Intensive	Mechanical	48 h	Left	Hyperacute	Clinical	7	Discharged	NAD
18	Goat	Saanen	3 y	Intensive	Mechanical	96 h	Right	Acute	Clinical	7	Discharged	None
19	Goat	Saanen	5 y	Intensive	Mechanical	15 days	Left	Chronic	Clinical	7	Discharged	Rigid MG
20	Goat	Crossbred	3 y	Intensive	Manual	24 h	Right	Hyperacute	Clinical	7	Discharged	Rigid MG
21	Goat	Saanen	2 y	Intensive	Mechanical	36 h	Right	Hyperacute	Surgical	30	Discharged	None
22	Goat	Saanen	5 y	Intensive	Manual	65 days	Both	Chronic	Surgical	87	Discharged	None
23	Goat	Saanen	6 y	Intensive	Manual	45 days	Both	Chronic	Surgical	62	Discharged	None

NAD: no available data. MG = mammary gland.

**Table 2:** Laboratory data from 23 small ruminants (16 goats and 7 sheep) with clinical mastitis.

Case	Ht	RBC	Hb	Leucocytes	SEG	Bands	LYM	MON	EOS	Fibrinogen	STP	Albumin	Globulin	Urea	CR	AST	GGT
1	34	18.8	11.3	7500	4275	-	3075	150	-	400	7.6	2.37	5.23	27	1.2	47	45
2	19	4.68	6.1	9100	7280	91	1547	91	91	600	6.4	1.25	5.15	30	1.4	165	7
3	40	18.7	12.2	2650	53	-	2518	53	27	400	5.1	2	3.1	130	2.3	146	45
4	35	13.3	10.9	3100	558	-	2201	310	31	1000	5.5	2	4.5	74	3.2	162	61
5 #	-	-	-	-	-	-	-	-	-	-	-	-	-	-	-	-	-
6	19	4.9	5.8	14,850	10,247	-	3119	149	1337	400	7.4	1.6	5.8	16	1.3	131	53
7	14	3.6	4	5200	3744	-	1300	156	-	400	5.2	1.3	3.9	30	1.1	172	84
8	29	8.1	9.1	15,100	11,300	-	3800	-	-	600	7.4	NP	NP	NP	NP	NP	NP
9	15	4.2	4.8	8700	5220	174	3306	-	-	800	6.8	NP	NP	NP	NP	NP	NP
10	17	8.8	5.7	13,050	9135	-	3132	653	131	400	10.7	1.7	9	23	1.5	47	-
11	24	11	6.1	8200	4592	-	2542	164	902	600	7.2	2.1	5.1	83	1.2	94	53
12	29	15.3	6.3	3050	702	-	2318	31	-	400	6.6	NP	NP	24	1.4	110	61
13	31	11.9	9.5	1800	612	-	1044	-	36	400	6	3.5	2.5	13	1.4	71	50
14	25	5,65	8	11,600	6264	-	1160	696	3480	200	7	2.8	4.2	34	0.7	109	72
15	29	7.9	6.4	7150	4219	-	1716	429	787	400	7.2	NP	NP	23	1.4	104	NP
16	22	10	7.1	13,210	10,701	133	2114	133	133	400	6.9	3.1	3.8	20	0.9	67	38
17	32	12.8	9	8650	6488	-	1990	173	-	1200	6.2	2.4	3.8	51	1.5	47	38
18	29	13.3	8.9	7000	3710	-	3080	140	70	400	6.4	2.9	3.5	23	1.3	89	30
19	20	12.3	7.2	8950	3849	-	4833	269	-	600	6	2.3	3.7	26	0.7	78	48
20	25	11.6	7.5	17,500	15,400	-	1925	-	-	200	6.2	1.7	4.5	49	1.1	115	122
21	34	16	10.8	3100	341		2449	279	31	800	5.5	2	3.5	67	1.7	178	30
22	24	10.9	7.7	5000	1300	-	2750	200	250	400	8.7	2.7	6	46	1.1	110	NP
23	30	12.9	9.7	8650	5709	-	2422	260	260	400	6.2	2.4	3.8	30	1.5	157	NP
X ± SD Goats	28.4 ± 6.2	13.1 ± 2.9	8.6 ± 2	7427 ± 4548	4495 ± 4453	133 ± 0	2559 ± 824	216 ± 155	187 ± 266	533 ± 269	6.7 ± 1.4	2.3 ± 0.5	4.4 ± 1.6	45.7 ± 31.6	1.4 ± 0.6	101.2 ± 43.6	51.7 ± 24.3
X ± SD Sheep	21.4 ± 6.2	5.5 ± 1.9	6.3 ± 1.7	8123 ± 4783	5433 ± 3471	132.5 ± 58.6	1833 ± 1305	304.2 ± 255.2	1423 ± 1462	485.7 ± 195.1	6.7 ± 0.7	1.7 ± 0.7	4.7 ± 0.8	26.6 ± 7.1	1.18 ± 0.3	136.2 ± 31.2	54 ± 33.8
RF Goats *	19–38	8–18	8–14	4000–13,000	1200–7200	0–100	2000–9000	0–650	50–650	100–400	6.4–7	2.7–3.9	2.7–4.1	21.4–42.8	1–1.8	43–132	20–56
RF Sheep *	24–50	8–16	8–16	4000–12,000	700–6000	0–100	2000–9000	0–750	0–1000	100–500	6–7.9	2.4–3	3.5–5.7	17.12–42.8	1.2–1.9	68–90	20–52

Ht: hematocrit (%); RBC: red blood cells (×10^6^/μL); Hb: hemoglobin (g/dL); SEG: segmented neutrophils (/μL); LYM: lymphocytes (/μL); MON: monocytes (/μL); EOS: eosinophils (/μL); STP: serum total protein (g/dL); CR: creatinine (mg/dL); AST: aspartate amino-transferase (UI/L); GGT: gamaglutamil transferase (UI/L); NP: not performed. RF: Reference values. * Meyer & Harvey [12], Kaneko et al. [13]. # The doe died shortly after clinical evaluation, and blood samples were not collected. X = mean; SD = standard deviation.

**Table 3:** Microbiological assays and in vitro antimicrobial susceptibility profile of milk samples from 13 small ruminants (10 goats and 3 sheep) with clinical mastitis.

Case	Affected MG	Isolated Bacteria	CEF	SUL + TRIM	AMOX	ENR	PEN	GEN	FLOR	CIPRO	AMP	TET	NOR	AZI
1	Right	No growth	-	-	-	-	-	-	-	-	-	-	-	-
3	Both	*Escherichia coli*	S	S	R	R	NP	NP	NP	NP	NP	NP	NP	NP
4	Left	*Staphylococcus aureus*	S	S	S	S	S	S	S	S	S	NP	NP	NP
6	Both	*S. aureus*/*E. coli*	NP	NP	NP	NP	NP	NP	NP	NP	NP	NP	NP	NP
7	Both	No growth	-	-	-	-	-	-	-	-	-	-	-	-
11	Both	*S. aureus*	S	S	R	R	R	S	NP	I	NP	S	NP	NP
12	Right	No growth	-	-	-	-	-	-	-	-	-	-	-	-
14	Right	*E. coli*	R	R	S	NP	NP	NP	NP	NP	S	R	S	R
	Left	*S. aureus*	S	R	S	NP	NP	NP	NP	NP	S	I	S	S
16	Both	*Streptococcus* sp.	S	S	S	S	R	S	NP	NP	R	S	NP	NP
18	Left	*S. aureus*	S	R	S	R	R	S	NP	S	S	NP	R	NP
	Right	*S. aureus*	R	I	S	R	R	S	NP	S	S	NP	I	NP
19	Left	No growth	-	-	-	-	-	-	-	-	-	-	-	-
20	Right	*Pasteurella* sp.	S	S	S	NP	R	S	NP	NP	S	NP	S	R
21	Right	*E. coli*	R	R	I	I	R	R	NP	S	R	R	S	R

MG: mammary gland; CEF: ceftiofur; SUL + TRIM: sulfadiazine plus trimethoprim; AMOX: amoxicillin; ENR: enrofloxacin; PEN: penicillin; GEN: gentamicin; FLOR: florfenicol; CIPRO: ciprofloxacin; AMP: ampicillin; TET: tetracycline; NOR: norfloxacin; AZI: azithromycin; S: sensitive; R: resistant; I: intermediate; NP: not performed.

## Data Availability

The original contributions presented in this study are included in the article. Further inquiries can be directed to the corresponding author.
